# A Strepsipteran parasite extends the lifespan of workers in a social wasp

**DOI:** 10.1038/s41598-021-86182-6

**Published:** 2021-03-31

**Authors:** Laura Beani, Romano Dallai, Federico Cappa, Fabio Manfredini, Marco Zaccaroni, Maria Cristina Lorenzi, David Mercati

**Affiliations:** 1 Dipartimento di Biologia, Università di Firenze, Via Madonna del piano 6, 50019 Sesto Fiorentino, Florence, Italy; 2 Dipartimento di Scienze Della Vita, Università di Siena, Via Aldo Moro, 53100 Siena, Italy; 3 School of Biological Sciences, University of Aberdeen, Aberdeen, UK; 4 LEEC, Laboratoire d’Éthologie Expérimentale et Comparée, Université Sorbonne Paris Nord, Villetaneuse, France

**Keywords:** Coevolution, Zoology

## Abstract

In social wasps, female lifespan depends on caste and colony tasks: workers usually live a few weeks while queens as long as 1 year. *Polistes dominula* paper wasps infected by the strepsipteran parasite *Xenos vesparum* avoid all colony tasks, cluster on vegetation where parasite dispersal and mating occur, hibernate and infect the next generation of wasp larvae. Here, we compared the survival rate of infected and uninfected wasp workers. Workers’ survival was significantly affected by parasite sex: two-third of workers parasitized by a *X. vesparum* female survived and overwintered like future queens did, while all workers infected by a *X. vesparum* male died during the summer, like uninfected workers that we used as controls. We measured a set of host and parasite traits possibly associated with the observed lifespan extension. Infected overwintering workers had larger fat bodies than infected workers that died in the summer, but they had similar body size and ovary development. Furthermore, we recorded a positive correlation between parasite and host body sizes. We hypothesize that the manipulation of worker’s longevity operated by *X. vesparum* enhances parasite’s fitness: if workers infected by a female overwinter, they can spread infective parasite larvae in the spring like parasitized gynes do, thus contributing to parasite transmission.

## Introduction

Parasite-induced alterations of host phenotypes have been described for a wide range of parasite-host relationships^1–3^. Indeed, many phenotypic traits of the host, from individual morphology and physiology to behaviour, can be affected by parasitic infection, and the magnitude of such alterations can greatly vary, from subtle changes in the percentage of time spent in performing a given activity to the production of complex and sometimes spectacular behaviours^4^.

A growing body of research on parasite-induced alterations of host phenotypes links the reduction in host lifespan to parasites draining resources and energy from their hosts [reviewed in^5^, as it occurs, for example, in honey-bee workers infected by *Varroa* mites and DWV viruses^6^ or bumble bees infected by tracheal mites^7^. In contrast, there are still few studies documenting the parasite capability to increase host longevity, a counter-intuitive effect of infection that enhances parasite’s survival. One such study reports that in *Tenebrio molitor* beetles infected by the rat tapeworm *Hymenolepis diminuta*, female’s lifespan increased by 40%^8,9^. The authors suggest that the parasite manipulates the host resource allocation and interferes with the trade-off between reproduction and longevity^10^, enhancing host survivorship and thus parasite transmission (see the model recently proposed by^11^).

Social insects provide an ideal opportunity to explore the mechanisms of increased host survival operated by parasites. In fact, lifespan in social insects naturally differs by caste, sex, and task performed in the colony^12^. Such a highly plastic trait can be exploited by parasites: sterile workers and males are short-lived (from few weeks to 2–3 months for temperate species), whereas queens are long-lived (several months to years, depending on the species). This is in contrast with the general assumption that a high reproductive effort is associated with reduced survival^13–15^. In primitively eusocial wasps like *Polistes* species living in temperate climates, morphological, physiological and behavioural differences between castes are not as pronounced as in highly eusocial wasps, bees and ants^16^. On the other hand, longevity can be strikingly different: mated gynes (i.e. next-year queens that are not yet egg-layers) overwinter and live up to 1 year, whereas workers and males typically live no longer than 30–40 days and die at the end of the summer^17–19^; although alternative life-histories have been described^20,21^.

The flexible caste determination mechanism of *Polistes dominula*^22,23^ is exploited by the strepsipteran *Xenos vesparum* (Xenidae), a parasitic castrator that usurps the host reproductive resources to complete its life cycle^24^. Parasitized *P. dominula* females, the primary host^25,26^, do not develop ovaries, desert the colony early in the season without performing any social task^27^ and forage on selected plants, rich of extra-floral nectaries secreting immune-stimulant compounds^28,29^. They form aberrant summer aggregations, where parasite mating occurs^30^, and may overwinter in sheltered sites with future queens^31^. Unlike the completely novel behaviour elicited by other manipulative parasites^32^, parasitized female wasps follow behavioural patterns and life-history trajectories that are still typical of the species, though usually confined to a specific caste: in fact, they all behave like gynes in non-reproductive phase^33,34^.

In line with the extreme sex-dimorphism of *X. vesp*a*rum* (Supplementary Materials, Fig. S1), male and female parasites differentially affect host survival. The twisted-winged adult male emerges from its puparium in the summer, inseminates a female and dies^30^, as does its host a few days after parasite emergence^26,31^. The neotenic endoparasitic female, a “bag” of oocytes and adipocytes, may instead overwinter within the host hemocoel. The female parasite is the reservoir of 1st instar larvae that can infect all larval stages of *P. dominula*^35^: these are the so-called triungulins, which are released on flowers or directly on nests by parasitized wasps^36,37^. The parasite cycle is bivoltine: there are two releases of triungulins, the first targeting worker wasps in the spring, the second targeting wasp sexuals in the summer^29^. Intriguingly, the effects of parasitic manipulation appear to depend on host sex; parasitized male wasps retain their sexual behaviour, die at the end of the summer and are absent from overwintering wasps’ aggregations^31^.

In a previous analysis of overwintering aggregations, the large variation in body size of parasitized wasps suggested that workers and gynes might cluster and overwinter together^31^. The overall goal of this study was to test the extended lifespan of parasitized putative workers, otherwise destined to die when colonies decline at the end of the summer, and investigate the underpinning mechanisms. If workers, infected by female parasites, are capable of overwintering like parasitized gynes do, the spreading of triungulins in the spring would be facilitated by a higher number of infected wasps: a potential adaptive outcome of parasite manipulation^2^.

To date, the survival of parasitized workers until the following spring has been only hypothesized^26,31,33,34^. Thus, in the present work, a substantial sample of workers were collected from flowering trumpet creepers in July (at a time when colonies do not yet produce sexuals), when parasitized wasps began to feed and aggregate on these bushes^28,29^, and were caged until March to assess their lifespan. We measured survival rates and a set of morpho-physiological traits (wasp size, abdominal lipid stores, ovary development) known to influence caste differentiation in *Polistes*^38^. Workers were either infected by a *X. vesparum* female or a male; we compared them—throughout the summer and hibernating period—to uninfected workers collected in the same period and to gynes collected later on (as controls)*.* Finally, we analysed how sex and body size of the parasite correlated with the morpho-physiological traits described above. Theoretical and empirical data^3,24^ suggest that parasitic castrators often grow to a body size that allows them to maximize their fitness without compromising host longevity.

## Results

### Host survival differs depending on *X. vesparum* sex and wasp caste

Monthly survival rates differed among the four groups of wasps that we analysed: workers parasitized by a *X. vesparum* female or by a male, non-parasitized workers and non-parasitized gynes, collected 3 months later, in October, when gynes form pre-hibernating aggregations (Table 1). Workers parasitized by a female had a peak of mortality between July and September (12 out of 34, 35%), but most of them survived until next spring (22 out of 34, i.e. 65%).

**Table 1:** Monthly number of live wasps (% survival rate) as a function of their caste and parasitic infection.

Monthly check	Workers parasitized by a *Xenos* female (n = 34)	Workers parasitized by a *Xenos* male (n = 30)	Workers non-parasitized (n = 21)	Gynes non-parasitized (n = 25)
July	33 (97%)	30 (100%)	20 (95%)	–
August	24 (71%)	22 (73%)	20 (95%)	–
September	22 (65%)	18 (60%)	14 (67%)	–
October	22 (65%)	15 (50%)	6 (29%)	25 (100%)
November	22 (65%)	4 (13%)	1 (5%)	25 (100%)
December	22 (65%)	4 (13%)	1 (5%)	22 (88%)
January	22 (65%)	1 (3%)	1 (5%)	21 (84%)
February	22 (65%)	0	1 (5%)	18 (72%)
March	22 (65%)	0	1 (5%)	14 (56%)

**Figure 1:**
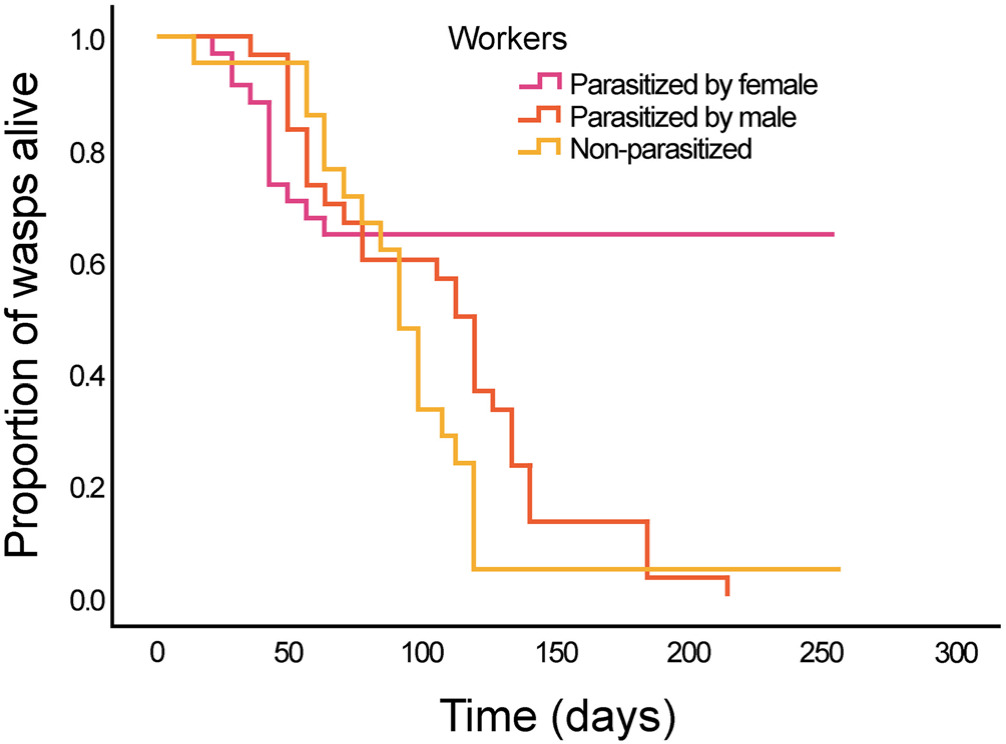
Survival rate of workers parasitized by a *X. vesparum* female (blue, n = 34), by a *X. vesparum* male (red, n = 30) and non-parasitized workers (green, n = 21) from July until March. Kaplan–Meier plot (Log Rank test, *p* < 0.001).

The daily survival rate among the three groups of workers (with female parasite, with male parasite and non-parasitized) was significantly different (Fig. 1, Kaplan–Meier test, *p* < 0.001) and mainly associated with the time of the year. While no difference in survival rates was observed in the first 3 months among workers (Table 2), post-hoc pairwise comparisons showed highly significant differences in the next months (encompassing winter). Workers parasitized by a female had a significantly longer lifespan than those parasitized by a male or non-parasitized at all (whereas workers parasitized by a male or non-parasitized did not differ in their survival rate and did not overwinter). The survival rate of gynes was lower than that of workers parasitized by a female (56% vs 65%), but higher than that of workers parasitized by a male and of non-parasitized workers (Table 2).

**Table 2:** Pairwise comparisons of survival rates between different categories of wasps.

Time period	Comparison	χ^2^	*p*
July–September	Workers infected by female versus male parasite	0.001	0.998
	Workers infected by female parasite versus workers non-parasitized	2.326	0.127
	Workers infected by male parasite versus workers non-parasitized	1.580	0.209
October–March	Workers infected by female versus male parasite	47.116	< 0.001
	Workers infected by female parasite versus workers non-parasitized	31.675	< 0.001
	Workers infected by male parasite versus workers non-parasitized	2.139	0.144
	Gynes versus workers parasitized by female parasite	12.330	< 0.001
	Gynes versus workers parasitized by male parasite	40.348	< 0.001
	Gynes versus workers non-parasitized	22.628	< 0.001

### Morpho-physiological differences among wasps in relation to parasitism, caste and lifespan

**Figure 2:**
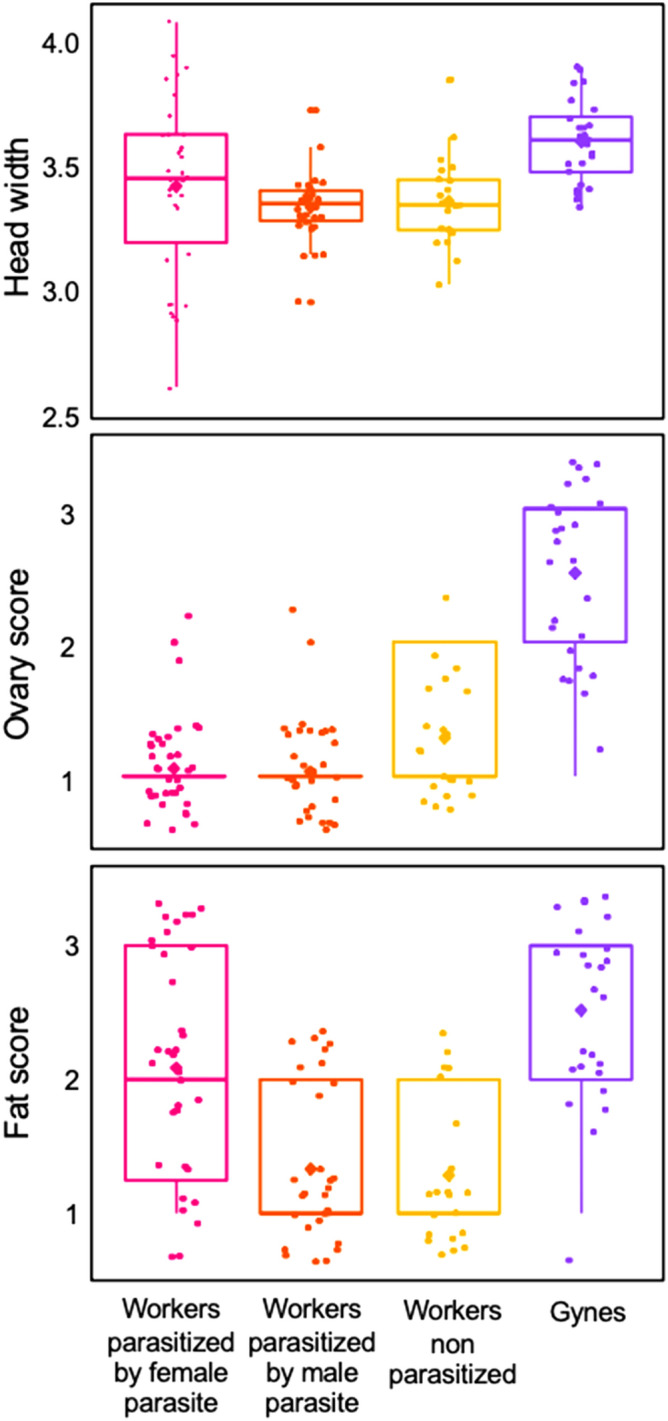
Head width (in mm), ovary score and fat score of workers parasitized by female or male parasites, non-parasitized workers and gynes. The boxplots show medians, quartiles, 5th and 95th percentiles and minimum and maximum values outside of the percentiles (color dots); diamonds represent means; points jittered to prevent overlap).

Host body size, measured as individual-head width^39^ (Fig. 2) did not significantly differ among workers infected by a *X. vesparum* female or male and non-parasitized workers (ANOVA F_2,82_ = 0.93, *p* = 0.39) despite the high sexual dimorphism and the different developmental trajectories of male and female parasites. As expected, gynes were significantly larger than workers (F_3,106_ = 6.49, *p* < 0.001), either non-parasitized (post-hoc Tukey’s pairwise test: *p* < 0.005) or parasitized by a female (*p* < 0.024) or a male (*p* < 0.005) parasite.

Ovaries in non-parasitized workers were more developed than in parasitized workers (Kruskal–Wallis: H = 2.7, *df* = 2, *p* > 0.001; post-hoc Tukey test: *p* < 0.02 and *p* < 0.001, female and male parasite, respectively), while there was no difference in ovary size in relation to parasite sex (Kruskal–Wallis: H = 0.03, *df* = 1, *p* = 0.63). As expected, gynes had higher ovary scores than parasitized workers (Kruskal–Wallis: H = 9.23, *df* = 3, *p* > 0.001; post-hoc Tukey test: *p* < 0.001).

With regards to fat storage, only gynes had visible masses of fat bodies and the difference among the groups was highly significant (Kruskal–Wallis: H = 38.64, *df* = 3, *p* < 0.001). Gynes had higher fat scores than non-parasitized workers (post-hoc Tukey test: *p* < 0.001) as well as workers infected by a male (*p* < 0.001), but this difference was less noticeable in comparison to workers infected by a female (*p* < 0.042). Infection of workers by a female parasite resulted in higher fat scores than in workers infected by a male or non-parasitized workers (*p* < 0.001).

With regards to the combined effect of lifespan and morphology, we compared the head width of 12 workers infected by a female *X. vesparum* that died between July and September, with that of 22 workers that survived until spring dissection, at the end of March. There was no significant difference in body size between these two groups (Student’s test, *t* = 1.31, *p* = 0.199), whereas the body size of 11 gynes that died before the winter was smaller compared to 14 gynes that survived until the spring (*t* = 2.65, *p* < 0.01). Ovary rank did not differ between spring-live and summer-dead workers (Mann–Whitney test: Z = 1.02, *p* = 0.309) as well as between spring-live gynes and those that died in the winter (Z = 1.5, *p* = 0.132).

**Figure 3:**
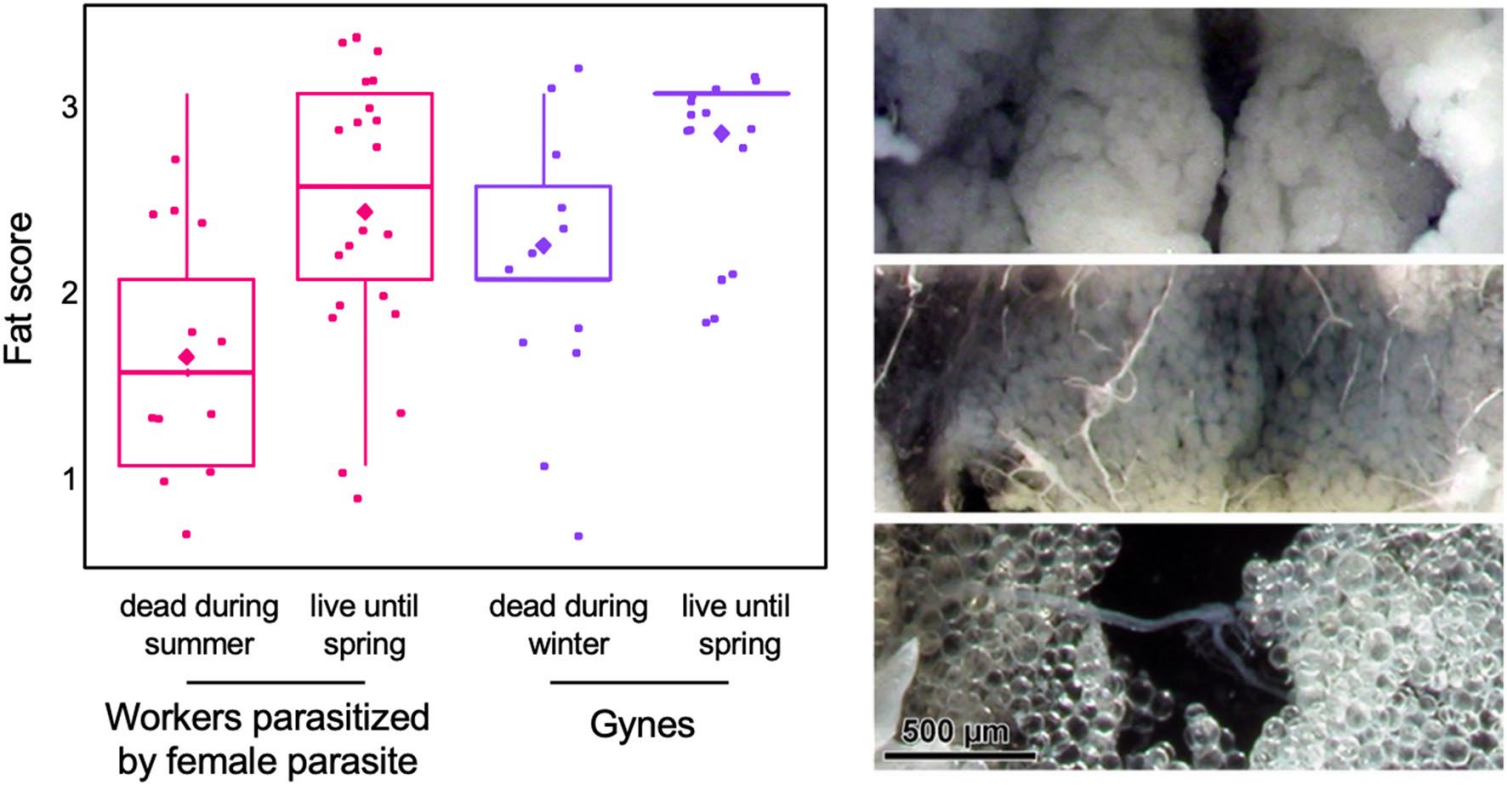
Fat scores of parasitized workers that died in the summer (n = 12) or survived until spring (n = 22) and non-parasitized gynes that died during the winter (October–February, n = 11) or survived until spring (n = 14). The boxplots show medians, quartiles, 5th and 95th percentiles and minimum and maximum values outside of the percentiles (color dots); diamonds represent means; points jittered to prevent overlap).

In contrast, overwintering parasitized workers (Fig. 3) had significantly higher fat scores than parasitized workers that died during the summer (Mann Whitney test: Z = 2.72, *p* < 0.006). The 14 gynes still alive in the spring had higher fat scores than the 11 gynes that died during the winter (Mann Whitney test: Z = 2.54, *p* < 0.01). No significant difference was recorded between the fat scores of 22 parasitized workers and 14 gynes, all of them overwintering (Mann Whitney test: Z = 1.81, *p* = 0.071). The fat bodies of 30 workers infected by a male parasite were mono-layered; these workers had lower fat scores than workers infected by a female parasite (Mann Whitney test: Z = 3.85, *p* < 0.001), while there was no significant difference with non-parasitized wasps (Mann Whitney test: Z = 0.34, *p* = 0.729).

In the photos, from the top: masses of fat bodies of a spring-live parasitized worker (score 3), pluri-stratified fat bodies of a winter-dead gyne (score 2), mono-layered fat bodies of a non-parasitized worker that died in summer (score 1).

#### Size matching between the host and the parasite

There was a strong body size dimorphism between *X. vesparum* males and females: females were about twice as big as males (Supplementary Materials, Fig. S1). Length (7.93 ± 0.12 mm) and width (2.48 ± 0.05) of *X. vesparum* females were measured for the whole sample (n = 34) while male puparia were measured for a smaller sample of workers that died between October and January (length 4.9 ± 0.03, width 1.66 ± 0.03, n = 7), as only in this sample males did not extrude from the puparium (in all other cases, the puparium was opened and destroyed at male’s emergence). Parasite length, ranging between 6.88 and 9.15 mm, and parasite width, ranging between 1.84 and 3.35 mm, were not correlated (Pearson’s r = 0.23, n = 34, *p* = 0.195).

**Figure 4:**
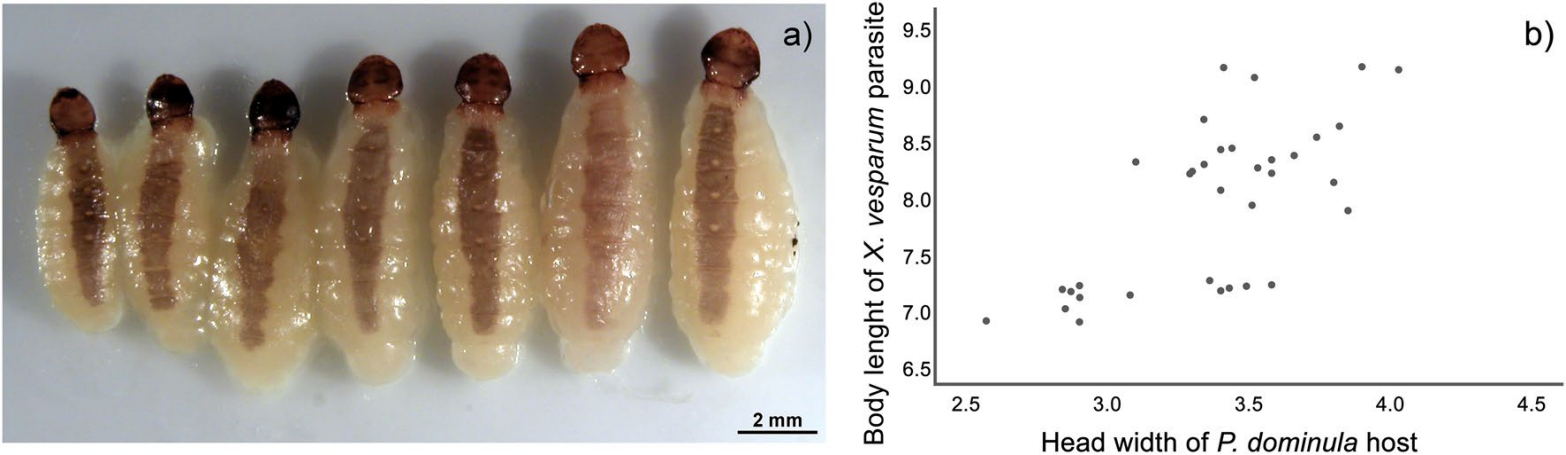
Size of *X. vesparum* females. (**a**) *X. vesparum* females dissected after the winter diapause (n = 7) sorted by size. (**b**) Correlation plot between host and parasite body size (n = 34). The head width of the wasps, ranging from 2.57 to 4.03 mm, is positively correlated with the length of the parasite (*p* < 0.001).

Female parasites were highly variable in body size (Fig. 4a). Intriguingly, parasite length, but not parasite width, was positively correlated with host body size (Fig. 4b) (Pearson’s correlation, length: r = 0.68, *p* < 0.001 *df* = 32; width: r = 0.054, *p* = 0.76, *df* = 32). The correlation between parasite length and host size remained significant if we considered only the sample of spring-live workers (r = 0.69, *p* < 0.001, *df* = 20) as well as the sample of summer-dead workers (r = 0.63, *p* < 0.03, *df* = 10), whereas parasite width was not correlated (*p* = 0.41 and *p* = 0.86, respectively). Unexpectedly, workers harbouring a larger *X. vesparum* female survived longer than workers infected by smaller parasites.

### Development of triungulins

**Figure 5:**
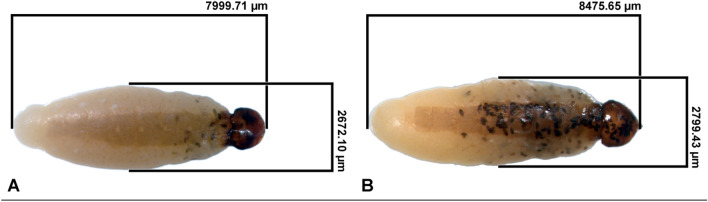
Two *X. vesparum* overwintering females containing few (**a**) and many (**b**) developed triungulins inside the brood canal. The amount of fully developed triungulins visible within the brood canal was variable, probably due to the presence of different developmental stages at the time of dissection.

Our experimental aggregation of wasps infected by both sexes of the parasite simulated semi-natural rearing condition that permitted the completion of the parasite life-cycle. In fact, 23 out of 30 *X. vesparum* adult males extruded from their puparia and successfully inseminated females: 24 out of 34 *X. vesparum* females, i.e. 71%, developed triungulins (Fig. 5). A first cohort of female parasites (8 out of 12), developed triungulins in summer 2018; a second cohort of female parasites (16 out of 22) developed triungulins the next spring. The length and width of female parasites were not significantly different between 24 females that produced triungulins and ten females that did not (respectively, Student’s test, *t* = 1.24, *p* = 0.22 and t = 0.32, *p* = 0.75), neither were the mean fat scores of the hosts (Mann Whitney test, Z = 0.32, *p* = 0.7). Unsuccessful fertilization, rather than parasite size or host condition, might affect the production of triungulins.

## Discussion

In the present study we provide the first evidence that the strepsipteran *X. vesparum* extends the lifespan of *P. dominula* workers, which can overwinter like future queens. The extended lifespan might enable workers harbouring a female parasite to become carriers of infective stages of the parasite the following spring. The factors underpinning this surprising shift in lifespan are: (1) the sex of the parasite, since workers infected by a male parasite die during the summer as uninfected workers do, whereas those infected by a female overwinter; (2) the amount of abdominal fat storage, more abundant in parasitized overwintering workers than in those dying during summer; (3) the positive correlation between parasite and host body sizes, which highlights a constraint in parasite growth. In summary, workers infected by a female parasite manage to overwinter instead of dying at the end of the summer as normally observed in temperate social wasps^14,40^, and by doing so they might enhance parasite transmission.

Parasite-induced extension of host lifespan, to date scarcely documented^8–10^, may be the result of parasite manipulation^41^ or simply a by-product of infection^1^. The unexpectedly high winter survival of two-third of *P. dominula* workers infected by a *X. vesparum* female (Fig. 1) provides more support for the manipulation hypothesis^2^, because the expanded lifespan of the host favors parasite transmission without enhancing host direct fitness: long-lived workers represent—together with overwintering infected gynes^42^—the spring reservoir of triungulins, developing after the host winter diapause and infecting host larvae in the spring. A third of the workers parasitized by a *X. vesparum* female died between August and September, at the time of the emergence of batches of triungulins from the parasite brood canal^26^: this also coincides with the time when the parasite targets the larvae of *Polistes* sexuals. Since the summer development of triungulins is variable across years, ranging from 15 to 100%^28^, the spring release of triungulins plays a critical role in parasite transmission to the next host generation.

The sex of the parasite differentially influences life-history and lifespan of the host^26,27^. This is further support for the parasite manipulation hypothesis. If larger parasites drain more nutrients from their hosts^43^, we would expect that workers harbouring large female parasites die earlier than those infected by small male parasites. In contrast, we found the opposite result, as the majority of the workers parasitized by large female parasites survived at least 6 months longer than those parasitized by males. In captivity, similarly to what observed in the field^28,29^, twisted-winged *X. vesparum* males emerged from their puparium, successfully fertilized females (71%) and died a few hours later. Workers harboring a male died in the summer, shortly after the emergence of the parasite (Fig. 1). They had undeveloped ovaries and lower fat scores than workers hosting a *Xenos* female, probably due to the costly development of a holometabolous insect (i.e. the male parasite), involving two further moults (compared to females) and the production of a puparium^30^. Noticeably, although for a small sample, wasps died later if *Xenos* males did not extrude from their puparium, suggesting that a hole in the puparium may facilitate microbial infections in the host^26,27^.

The parasite exploits the flexible lifespan and caste system of the primitively eusocial *P. dominula*. Previous research has shown that workers emerged from young colonies and transferred to mature colonies, at the stage when future queens are produced, show an extended lifespan and, at least in the lab, do not perform any colony task and behave like future queens^44^. Moreover, a relevant fraction of the first-brood workers may adopt an alternative reproductive strategy, called the sit-and-wait tactic: they leave their nest early, overwinter and found a colony the following season^20,21^. In captive conditions without environmental hazards^52^, non-parasitized workers survived less than 100 days from their collection at the beginning of July. Foraging activity before capture was unlikely to be cause of early mortality. In fact, foraging usually causes wing wear in flying insects^38^ and our workers showed no sign of wing wear.

Parasitized wasps desert the colony without performing any colony task, similarly to gynes^27^, thus avoiding the extrinsic mortality factors related to costly and risky duties, such as brood rearing, foraging for the colony, nest building and defence. Plausibly, the parasite shifts the plastic expression of caste-related genes in workers, eliciting gyne-like behaviour and up-regulation of the immune-response protein 30 (IRP30) and the antimicrobial peptide Defensin^34,45^. Despite the possible survival costs associated with the activation of the immune system in response to a parasite^46^, 65% of workers infected by a *Xenos* female survived until the experimental dissection at the end of March. The altered feeding frequency^28,29^ of parasitized wasps on trumpet creepers, source of our experimental sample, might be a compensatory behavior that increases the uptake of nutrients and immune-stimulating substances^47^. Indeed, a key factor for winter survival is resource storage^48–50^, as fat bodies release energy during prolonged non-feeding periods as hibernation^51^. Notably, among the morphological features that we measured, only abdominal fat storage was associated with longevity of overwintering workers, while body size and ovary rank did not. Workers infected by a *Xenos* female survived if they store up fat bodies like gynes that are alive at the end of March, whereas those with low fat scores died at the end of summer. Previous data on fat bodies in parasitized and non-parasitized wasps dissected in August and September^31^ were comparable to the current data of wasps that died in the same months. Therefore, the scarcity of fat bodies detected in these wasps is not an experimental artifact due to the fact that dissections were performed after wasps had died. The increased investment in fat body by overwintering parasitized workers might be triggered by enhanced expression before overwintering of caste-dependent genes associated with lipid metabolism^34^. Indeed, it has been reported in *P. metricus* that gynes invest heavily in energy storage as an adaptation to overwintering^38^.

According to Hamilton^52^, *X. vesparum* developed the ability to castrate *P. dominula* wasps “without killing them” during a long coevolutionary process; not surprisingly, this parasite might be able to lengthen host lifespan by matching its development with host growth. We found a significant correlation between host size and female parasite length, in line with a previous study on Strepsiptera^53^: the size of female parasites is smaller when they parasitize small male wasps or workers than when they parasitize large hosts such as gynes and queens. The correlation between host and parasite body size is known as Harrison’s rule, well documented in parasitized birds^54–56^ but rarely investigated in insects. In the *X. vesparum*-*P. dominula* system, size matching may be due to the quantity of nutrients processed by both the host and by its female endoparasite during host larval development, because after wasp emergence the parasite stops depleting host resources^55^. This subtle strategy of tuning to the host size, typical of parasitic castrators^3,24^, allows them to limit the costs of parasitism. In this perspective, a perfectly developed parasite can enhance host survival and prolong its lifespan. Workers harbouring larger *X. vesparum* females—in width and length—survived until spring, i.e. longer than workers infected by smaller parasites. Thus, the size of *X. vesparum* females increases along with host size, taking up all available abdominal space but avoiding the risk of resource depletion and without compromising host survival: indeed, there was no significant size difference between parasitized and non-parasitized workers. Moreover, the development of triungulins confirmed that the laboratory conditions did not compromise the parasite life cycle.

In conclusion, we report here a novel manipulation strategy, hypothesized but not tested before. Parasite fitness increases by exploiting the resource of castrated wasps and by expanding their lifespan: a trade-off between reproduction and longevity faced by parasitic castrators^24^. Strepsiptera might be defined macrynobionts (*macryno*, lengthen; *bios*, life), as proposed by Kathirithamby^26^. The lifespan of parasitized workers may be significantly extended—in overwintering workers—or preserved—in workers that died in summer after the release of triungulins—depending on the parasite interests, but never reduced: thus, workers may act as summer and spring reservoirs of triungulins. In the scenario of the long coevolution history between *X. vesparum* and *P. dominula*, the parasite affects a wide range of host traits, from behavior to physiology and lifespan: at any level of analysis, parasitized wasps are the extended phenotype of the castrator^24^.

## Methods

*P. dominula* workers were sampled in early July on two trumpet-creepers bushes (*C. radicans*) in the plain of Sesto Fiorentino (Florence, Italy: 43° 50′ 7″ N, 11° 11′ 46″ E). As we lack clear-cut morphological cues for caste determination in this species^16^, we used time of sampling to predict wasps’ putative caste: wasps that emerge until mid-July are normally workers^17,18^, while wasps that emerge from August onward are usually sexuals, i.e. gynes and males, and colonies are proterandric in the production of reproductive individuals^56^. The absence of *P. dominula* males in our collections from trumpet-creepers bushes supports therefore our assignment of sampled wasps to the worker caste. We collected a sample of 89 workers: 35 putative workers infected by one *X. vesparum* female, 31 workers infected by one *X. vesparum* male and 23 non-parasitized workers. As the extrusion of parasites—cephalothorax if female, puparium if male—occurs 1–2 weeks after wasp emergence, we were able to identify the parasite sex and remove from the experiment any wasp with 2 or more parasites to avoid the confounding factor of multiple parasites draining resources from the same host. Moreover, in mid-October, we collected a pre-hibernating cluster of 26 non-parasitized wasps in the same area; these wasps were classified as gynes based on the time of collection, and their size and behaviour^56^. Gynes were not included in survival analysis, due to the late collection date, 3 months after workers. Non-parasitized workers and pre-hibernating non-parasitized gynes were used as controls. Since parasitized wasps desert their colonies before parasite extrusion, it was not possible to assess the colony of origin.

Wasps were housed in a large cage (50 × 50 × 50 cm) from 4th July 2018 until spring, exposed to natural light and temperature (range: 5–28 °C), and supplied with sugar and water ad libitum. Wasps were marked with individual Testor’s paints on the thorax to distinguish workers infected by a parasite female from those infected by a parasite male, or non-parasitized workers and gynes. We weekly removed dead wasps from the cage and froze them. At the end of March, we froze all wasps that survived and blind-dissected them (i.e. without a priori knowledge of their survival state) to measure morpho-physiological traits^37^. We excluded from the analysis 5 wasps that were not adequately preserved. Overall, we recorded lifespan and described morphology of 110 wasps. As we aimed to measure longevity and morphological traits in the same individuals, it was impossible to score fat bodies in live wasps, as this would have required killing the wasps. The choice of the end of March as the conclusion of the survival experiment is consistent with field observations, which report that wasps leave overwintering aggregations in April, when new nests are founded^31^.

Wasps and their parasites were photographed using a digital camera (Zeiss MRC5) and a stereomicroscope Olympus. Pictures were used to measure female head width (a reliable indicator of body size^39^, parasites’ body size (length and width, see Fig. 4), and to evaluate the occurrence of mature triungulins inside *X. vesparum* females. We evaluated ovarian development as score 1 if undeveloped thread-like ovaries were observed, score 2 if ovaries contained detectable oocytes and score 3 if ovaries contained mature oocytes. We evaluated fat bodies under the third tergite (see Fig. 2) and ranked them score 1 if monolayered, 2 if multi-stratified and 3 if we observed masses of adipocytes (see Fig. 2 and^31^ for details).

### Statistical analysis

All statistical analyses were conducted using PAST3^57^. Differences in daily survival were analyzed using Kaplan–Meier test (χ^2^ test for pairwise comparisons). Descriptive statistics were given as means  SE. We used parametric tests to analyze the body size of wasps and parasites (Student’s *t* test, ANOVA and Tukey test as a post-hoc test), after checking for normality and equality of variance (Shapiro–Wilk test). We tested for correlation between host and parasite body size using Pearson correlation test. We used non-parametric tests to analyze fat score and ovary score (Mann–Whitney U test, Kruskal–Wallis test, χ^2^ test). Bonferroni correction for multiple comparisons was applied, setting significance level at 1.7%.

### Ethic statement

Collection of live wasps and dissection of frozen samples comply with the current laws in Italy. The species used in the experiments (*P. dominula*) is not endangered or protected in Italy.

## Supplementary Information

Supplementary Information
