# Supplementary material for: A Strepsipteran parasite extends the lifespan of workers in a social wasp
Source: Sci Rep. 2021 Mar 31;11:7235. doi: 10.1038/s41598-021-86182-6 (PMC8012566; doi:10.1038/s41598-021-86182-6)
Supplement: Supplementary file 1 — Supplementary Information [file 41598_2021_86182_MOESM1_ESM.pdf]

A Stresipteran parasite extends the lifespan of workers in a social wasp

Laura Beani[1*], Romano Dallai[2], Federico Cappa[1], Fabio Manfredini[3], Marco Zaccaroni[1], Maria Cristina Lorenzi[4], David Mercati[2]

Supplementary material

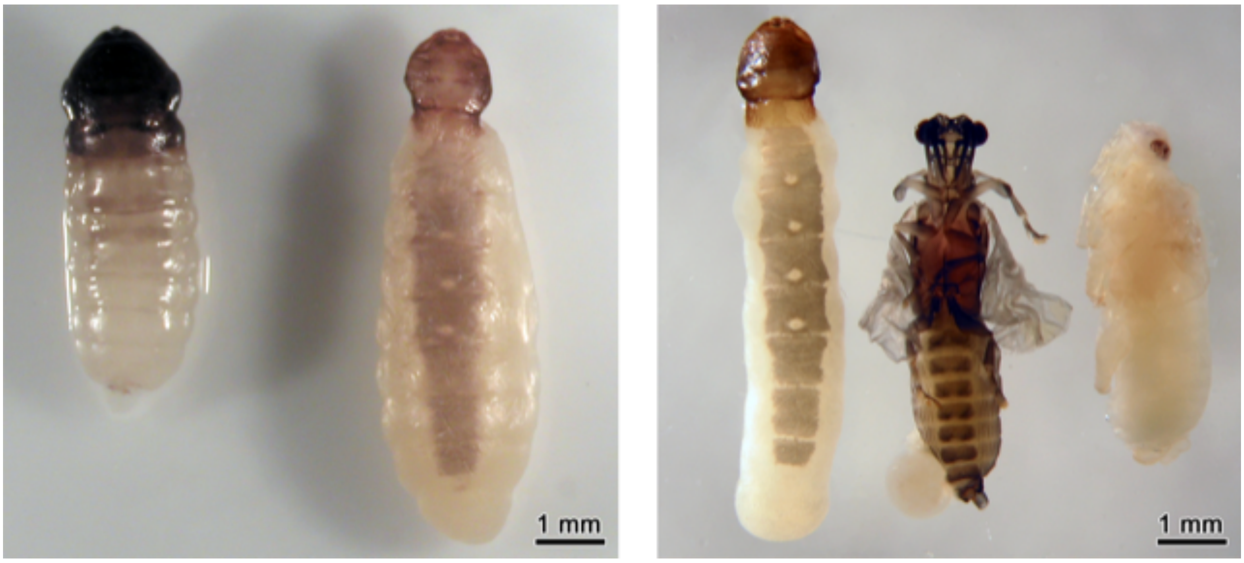

Fig. S1– Male and female *X*. *vesparum* parasites. Left: a male puparium and a neotenic female. Right: a female, a male imago and the last pupal stage.
